# Past, current and future

**DOI:** 10.17712/nsj.2017.1.20170006

**Published:** 2017-01

Two decades ago, professors Saleh M. Al-Deeb, Khalaf Al-Moutaery and Basim Yaqub launched the neurosciences bulletin as the specialized journal in the kingdom and the Arab world due to the growing interest in neurosciences research and publication. The journal started as the official bulletin of the Neurosciences Department, Prince Sultan Military Medical City (PSMMC) as a twice-yearly publication including articles, and abstracts from related meetings. After that, it became a quarterly publication, and continues to be well-respected peer review journal and services its mission as a mean to disseminate accessible, up to date medical information for clinical neurosciences and promote neurosciences research locally and internationally.

With our devoted and hardworking editorial team the journal achieves successful steps and improvements. In 2000, the journal established the journal website which now had 124,813 hits, where readers can view, read and download the articles PDF. In 2006 the journal received an approval for indexing by the Thomson Scientific (formerly known as Thomson ISI) and following that the journal indexed and included in MEDLINE and that the English abstracts will be included using PubMed in July 2010. In 2016, we start including the journal in the Pub Med Central, and increasing the journal visibility in many databases.

**Figure 1:**
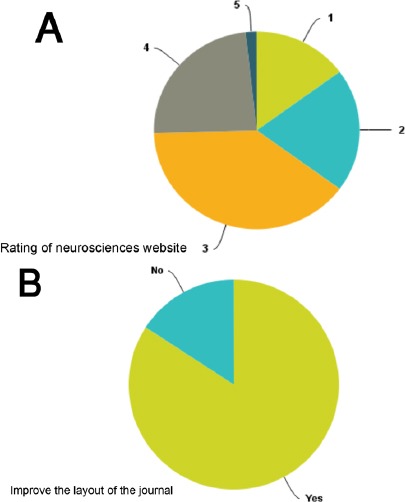
- A pie chart Illustrates **a)** the rating of neurosciences website 1 represent the lowest score and 5 represents the highest score, **b)** the need to improve the layout of the journal

After years of dedicated editorship, the journal associate-editors Dr. Sonia khan and Dr. Mohammed Al-Zawahmah left the journal in 2016 after many accomplishments. In this regard, we would like to introduce ourselves to the readers as associate editors of neurosciences journal. Dr. Waleed Khoja is Deputy Director of the Neurology at the PSMMC; founder and past president of Saudi Stroke Association. Dr. Khaled hundallah is pediatric neurology consultant at the PSMMC and assistant professor at Al-Imam Muhammad Ibn Saud Islamic University.

To set our strategic plan, we decided to involve our target audience. An electronic survey was distributed and posted in the journal website. We received a total of 120 respondents. More than 60% of our respondents spend 2 hours and more weekly reading the medical journal and 65.83% find the journal important in their field. For the journal website, 39.47% of the respondents rate the journal website 3 out of 4. For the journal logo, 55% find out the logo represents the journal. However, improving the journal website, decreasing the processing time and establishing an electronic submission system, and increasing the journal visibility are the key points our audience look for.

To satisfy the needs, demands and the expectations of our target audience the following steps were initiated. To increase the visibility of the journal, we invite all the local societies related to the neurosciences to sign an agreement and the journal will be the official journal of the society. In return, the society will contribute to the development of the journal scientifically. We also focus on the student corner and encourage the students to submit and publish an abstract of their research. The students’ abstracts will be published in the journal website and indexed in the pub med central. In addition to that, e-journal, the journal website, old issues archives, online submissions systems are all areas in our agenda.

As per to a local study conducted by Jamjoom^1^ during the period 1996-2014 to evaluate the sate of the clinical Neurosciences research in Kingdom of Saudi Arabia by determining its world ranking finds out that KSA is ranked 14th in the world and fourth in the middle east on clinical Neurosciences during the period 1996-2014. The study shows that KSA was ranked low in the world. This finding would suggest a strong action to promote research in our region and encourage the neuro clinicians in this field.

In 2016, we received a total number of 135 manuscripts, with an average rejection rate of 37.8%. Reasons for rejection included papers out the scope of the journal, of low scientific quality, not meeting the requirements of the journal, authors failing to submit the revisions and other necessary requirements, and duplicate publication. However, over 4 issues of 2016, we published a total number of 70 articles which include: 22 originals, 1 editorials, 9 reviews, 11 case reports, 10 clinical note, 3 brief communications, 2 brief report, 4 correspondences, 1meeting highlight, and 4 MCQs. The average processing time from received to acceptance was 2.6 (1-6) months and 3.1 (1-9) months from acceptance to publication and we are pleased to decrease the average processing time frame this year.

**Figure 1:**
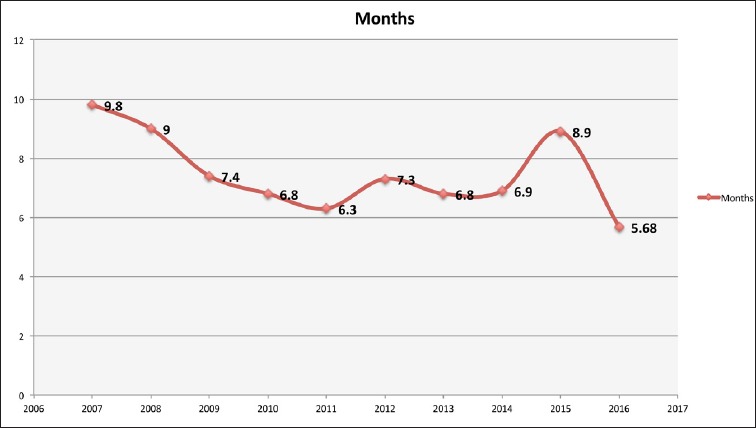
- Illustrates the processing time from submission to publication

We extend our thanks to the Editorial and Advisory Board Members for their significant contributions to maintaining the standards of the journal, and we look forward to their important continued role in achieving our goals for 2017. We would like to thank the outgoing Advisory Board member (Saleh Baeesa) who had now finished his term and would like to welcome Nader Honjol to the Advisory Board. We also extend our thanks to the outgoing Editorial Board members (Ahmad Khalifa). We are honored they have agreed to join the board, and their individual contributions will be of great value to the journal. We continue the international diversity of members that the current board offers. We hope all our readers continue to benefit from the published material, and we extend our sincerest thanks to our authors, readers, reviewers, and board members, and wish all a successful year.

Waleed Khoja

***Associate Editor***

Khalid Hundallah

***Associate Editor***

## 

*Our thanks also go to the following reviewers, who have participated in the excellent review of manuscripts and books for the year 2016*.

Angela Vincent

Adria Arboix

Adria Arboix

Ahmed H El Beltagi

Ahmed Osman Abdel-Zaher

Ajay Panwar

Alberto Verrotti

Alberto Verrotti

Ali Hassan Rajput

Alim P. Mitha

Annalisa Pastore

Boyd Koffman

Burak Karaaslan

Carlotta Spagnoli

Celia Godfrey

Chetan G K

Christian Brandt

Christian Pagnoux

Christian Urbanek

Clement Darling

Dan Harrison

Danielle Rice

Elena Erro Aguirre

Elena Zapata-Arriaza

Elisa Tamburo

Emiel Cracco

Emilio Perucca

Erling Tronvik

Ernane Torres Uchoa

Ezequiel Uribe

Fabrizio Schifano

Fahad Abdu Bashiri

Fatih Çelikel

Feng Zhang

Flávia de Lima Osório

Gro Solbakken

Hadeel Al Khamees

Hai Zhang

Hermann Stefan

Hiroshi Kataoka

Hiroyuki Kobayashi

Hsiuying Wang

Hung-Yu Chan

Ioannis Karakis

Jaime Gállego Culleré

James Siegler

Jaume Roquer

Javier Pablo Hryb

Jermaine dambi

Jian-min Liu

Jinn-Rung Kuo

José Roberto Wajman

Joseph Jankovic

Jun Zhang

Junshi Wang

Katrin Hahn

Kenneth Liu

Konark Malhotra

Leonie Walker

luca Gallelli

Luigi Maria Cavallo

Luisa Escobar Sánchez

Lynette M. Silva

Majed AlHameed

Manonita Ghosh

María Frenzi Rabito Alcón

Marjo van der Knaap

Martha Eva Viveros-Sandoval

Martha Shiell

Masahito Mihara

Matias Noll

Matti Isokangas

Meheroz H. Rabadi

Michael Bergin

Michael G. Young

Michael Ssonko

Miguel Angel Palomero Rodríguez

Mirjana Babic Leko

Mohammad Tariq

Mohammed Hasan Bangash

Mohammed Jan

Myoung Soo Kim

Nadia El Kadmiri

Nisa Cem Ören

Oktay Algin

Ove Sonesson

Pasquale Striano

Patricia Rzezak

puneet mittal

R.Van den Berg

Raidah Saleem Albaradie

Raúl Pelayo

Rayaz Malik

Richard Bright

Riyadh alokaili

Robert Dumaine

Rohan.R.Mahale

Roland Thijs

Samir R. Belagaje

Sandeep Talari

Sandra Orozco–Suárez

Sanja Pekovic

Seyed Ali Nabavizadeh

Shanshan Jiang

Slaven Pikija

Sotirios Giannopoulos

Sushil Sharma

Suzannah Iadarola

Tayfun Hakan

Thomas Hays

Tracy Melzer

Wolfgang Herrmann

Wouter Vanderplasschen

YuBao Jiang

Yun Hwang

Zaida Chinchilla-Rodríguez

Zhongheng Zhang
